# Editorial: Antimicrobial resistance: agriculture, environment and public health within One Health framework

**DOI:** 10.3389/fmicb.2023.1252134

**Published:** 2023-11-01

**Authors:** Tao Li, Haihong Hao, Xiaolin Hou, Jiang Xia

**Affiliations:** ^1^Shanghai Veterinary Research Institute, Chinese Academy of Agricultural Sciences (CAAS), Shanghai, China; ^2^China MOA Laboratory for Risk Assessment of Quality and Safety of Livestock and Poultry Products, Huazhong Agricultural University, Wuhan, China; ^3^Beijing Key Laboratory of Chinese Veterinary Medicine, Department of Veterinary Medicine, National Demonstration Center for Experimental Animal Education, Beijing University of Agriculture, Beijing, China; ^4^Department of Chemistry, School of Life Sciences, The Chinese University of Hong Kong, Shatin, Hong Kong SAR, China

**Keywords:** antimicrobial resistance, agriculture, public health, One Health, ecosystem, drug resistance

Recently, due to the increasing spread of antimicrobial resistance (AMR), AMR has received great attention from researchers and governments. It is expected that AMR will be the most important public health issue and challenge in this century (Nahrgang et al., 2018). Infections caused by AMR bacteria are arguably more serious and less responsive to treatment than infections caused by sensitive bacteria. Infections caused by AMR bacteria lead to consequences ranging from treatment failure and the need for more complex treatments to increased morbidity and mortality rates, hospitalization, and healthcare costs (Chinemerem Nwobodo et al., 2022). Therefore, AMR requires urgent multisectoral action in order to achieve the Sustainable Development Goals (SDGs).

Many studies have demonstrated that AMR is an ecological problem, as most bacteria and their antibiotic resistance genes (ARGs) can quickly move between humans, agriculture, and the environment (Bottery et al., 2021; Wang et al., 2023), creating a serious problem. Along with humans and the environment, the One Health framework also encompasses agriculture/plant sciences due to the importance and interdependence of AMR in relation to public health, the environment, and animals.

This Research Topic focuses on recent studies on the impact of AMR on human and/or animal health and innovative technologies and strategies to combat AMR.

## AMR in the ecosystem

As mentioned above, ARGs circulate among the environment, agriculture, and humans, which represent the different components of the One Health framework. In ecological processes, soil bacteria play an important role and are highly influenced by various agricultural activities, which impact the chemical, macroscopic, and microbiological composition. Cardenas Alegria et al. analyzed organic soil from various forests, pastures, and transgenic soybean monocultures in Brazil. They found diverse ARGs in forest soils, and especially in soybean crop soils. The most common ARGs were those related to aminoglycosides, macrolides, tetracyclines, and fluoroquinolones. Zhang et al. found that certain feces-derived ARGs (such as *aadA1* and *bla1*) could be easily transferred from paddy soil to field water. Similarly, Han et al. reviewed recent studies on the associations of ARGs with microbiomes and the mechanisms of ARG dissemination in soil. They found that the presence of ARGs in soil is inherent and ancient, and plants and animals can absorb ARGs from the soil and then transmit them to humans. Also, Gibson et al. were the first to analyze the impact on AMR in wastewater treatment plants of the effect of microbial immigration on the indigenous resident microbial communities.

Wild birds, which are an integral part of the ecological system, have been assessed as possible reservoirs and disseminators of ARGs to insular biomes in experimental studies, but their potential role as dispersers under real-world conditions remains unknown. Island settings provide a unique opportunity for scientists to evaluate and debate the spread of ARGs by wild birds in the ecological system. Ewbank et al. evaluated cloacal swabs from wild *Fregata magnificens* in the Alcatrazes Archipelago, which is a nature reserve without any human occupation before. They found a highly virulent multidrug-resistant (MDR) ST648 (O153:H9) pandemic *Escherichia coli* clone, which is one of the most commonly reported international sequence types (STs) in the global human–animal–environmental interface. Alcatrazes *Fregata magnificens* most likely have extremely limited to no direct contact with humans, due to their site fidelity and limited roosting and nesting area on the island. The authors speculated that a route of infection of Alcatrazes *Fregata magnificens* by the abovementioned *E. coli* strain may involve their continuous close contact and body fluid exchange with other frigatebirds and birds of other genera [especially (*Sula leucogaster* and *Coragyps atratus*)] using the area.

Shrestha et al. evaluated the association between antimicrobial use in turkey flocks and the development of AMR in North America. Using the indicator bacterium *E. coli*, they reported the effect of the antimicrobial treatment for certain diseases (such as enteric diseases, advanced sepsis, and Colibacillosis) on the development of resistance to certain antimicrobials. they concluded that from an antimicrobial management perspective, interventions at the producer level may have the greatest impact on reducing AMR establishment in *E. coli* isolates.

In recent years, there has been increasing attention on the association between gut dysbiosis and AMR. Gut dysbiosis contributes to the loss of colonization resistance, which can be followed by increased AMR. However, research has mainly focused on the effect of antibiotics on the gut microbiota, while ignoring the effect of other drugs such as antiparasitic drugs. The effects of salinomycin and ethanamizuril (which represent two different classes of coccidiostat, and are widely used in China) on the microbiota of cecal contents, manure compost, and soil were unknown. Cheng et al. compared the effects of these two treatments on the microbiota of coccidia-infected broilers' cecal contents, manure compost, and loam soil. They found that both treatments suppressed certain opportunistic pathogens, but they failed to repair the large changes in the cecal microbiota caused by coccidia infection. The *in vitro* effect of ethanamizuril on compost and soil microbiota seemed to be slight, while salinomycin had a large impact on these microbial communities.

## AMR prevention and control

This Research Topic also focuses on how to prevent and control AMR. Natural product extracts are increasingly being recognized as alternative antimicrobials for the treatment of infectious diseases in animals. Gu et al. reported that geraniol, the main component of various essential oils, not only effectively inhibits the formation of methicillin-resistant *Staphylococcus aureus* biofilms, but also relieves inflammatory symptoms and potentiates vancomycin by destroying biofilm structures. There is evidence that the activity of conventional antibiotics can be enhanced when used synergistically with natural product extracts. Breser et al. studied the effect of cloxacillin and chitosan alone or in combination on non-*S. aureus* isolates with various lifestyles and biofilm-forming abilities. They found that the addition of chitosan to the cloxacillin treatment significantly reduced the bactericidal concentration of the antibiotic, irrespective of the biomass density of the biofilm or the AMR pattern of the non-*S. aureus* isolates.

## AMR diagnostics

Rapid detection of AMR and prediction of future AMR burden and trends are crucial for implementing appropriate interventions to mitigate AMR. Several studies have used various machine learning algorithms to predict AMR rates using data on phenotypes and genotypes. Kim et al. collected 2010–2021 pathogen and AMR data from >600 farms in the United States to conduct AMR time series analyses using machine learning. They established three machine learning-based time series analyses to predict the future AMR rate. They found that the seasonal auto-regressive integrated moving average (SARIMA) approach had low root mean square error compared to other approaches, and it worked even for highly dynamic time series.

Whole-genome sequencing plays an extremely important role in AMR research, and it has provided insights into AMR mechanisms, pathogen evolution, and population dynamics in global pathogen surveillance. Zhou et al. found that 87.85% of *E. coli* isolates from chicken and pigs had MDR (the main MDR pattern was AMP-SPT-TET-FFC-SF-SXT) and there were 65 sequence types (STs) among the *E. coli* isolates. Yang et al. screened a total 921 samples from pigs and humans in China for the plasmid-encoded *tet(X4)* ARG (which mediates tigecycline resistance and has been widely identified in *E. coli* isolates from food-producing animals). They isolated two *tet(X4)*-positive strains, and conjugation experiments showed that the two *tet(X4)*-encoding plasmids could be transferred from *K. pneumoniae* to *E. coli*.

In conclusion, we believe that the data collected in the aforementioned studies and reviews can provide physicians, veterinarians, environmental scientists, public health professionals, wildlife experts, and many others with a toolkit for use within the One Health framework.

## Author contributions

All authors listed have made a substantial, direct, and intellectual contribution to the work and approved it for publication.
